# Effects of truncations in the N‐ and C‐terminal domains of filensin on filament formation with phakinin in cell‐free conditions and cultured cells

**DOI:** 10.1002/2211-5463.13700

**Published:** 2023-08-30

**Authors:** Moe Tashiro, Akari Nakamura, Yamato Kuratani, Miyako Takada, Satoshi Iwamoto, Mikako Oka, Shoji Ando

**Affiliations:** ^1^ Faculty of Biotechnology and Life Science Sojo University Kumamoto Japan; ^2^ Faculty of Pharmacy Keio University Tokyo Japan; ^3^ Present address: Yokohama University of Pharmacy 601 Matano‐cho, Totsuka‐ku Yokohama 245‐0066 Japan

**Keywords:** beaded filament, cataract, filensin, fluorescent protein, intermediate filament, phakinin

## Abstract

Filensin and phakinin are lens fiber cell‐specific proteins that constitute the beaded filaments (BFs) that are critical for maintaining lens transparency. In the Shumiya cataract rat, filensin 94 kDa undergoes N‐ and C‐terminal proteolytic processing to give a transient 50 kDa fragment and a final 38 kDa fragment, just before opacification. To characterize the effects of this processing on filensin function, recombinant proteins representing the two filensin fragments, termed Fil(30–416) and Fil(30–369), respectively, were examined. Fil(30–416) lacks the N‐terminal 29 amino acids and the C‐terminal 248 amino acids. Fil(30–369) lacks the N‐terminal 29 residues and the C‐terminal 295 residues. In cell‐free assembly characterized by electron microscopy, filensin and Fil(30–416) co‐polymerized with phakinin and formed rugged, entangled filaments, whereas Fil(30–369) formed only aggregates. In cultured SW‐13 and MCF‐7 cells expressing fluorescent fusion proteins, filensin and Fil(30–416) co‐polymerized with phakinin and formed cytoplasmic sinuous filaments with different widths, while Fil(30–369) gave aggregates. Therefore, while truncation of the N‐terminal 29 amino acids did not affect filament formation, truncation of the C‐terminal 295 but not the 248 residues resulted in failure of filament formation. These results indicate that the tail B region (residues 370–416) of rat filensin is essential for filament formation with phakinin. Truncation of the tail B region by proteolytic processing in the cataract rat lens might interfere with BF formation and thereby contribute to opacification.

## Graphical abstract

Filensin (Fil) and phakinin (Phk) are lens‐specific proteins that constitute beaded filaments, which are critical for maintaining lens transparency. To characterize the effects of proteolytic processing on filensin function in the Shumiya cataract rat lens, two recombinant proteins, Fil(30–416) and Fil(30–369), representing filensin fragments, were examined. The filensin tail B region was shown to be essential for filament formation with phakinin.

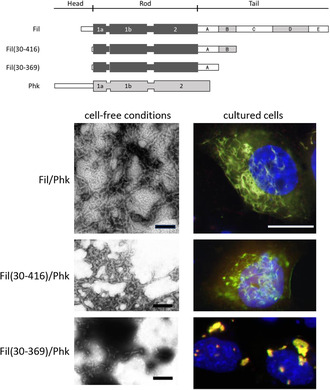

**Abbreviations**
AcGFP: *Aequorea coerulescens* green fluorescent protein
BF: beaded filament
DAPI: 4′,6‐diamidino‐2‐phenylindole
HIM: helix initiation motif
HTM: helix termination motif
IF: intermediate filament
mCherry: monomeric Cherry fluorescent protein
SCR: Shumiya cataract rat

Lens fiber cells contain beaded filaments (BFs), cytoskeletal elements that are structurally distinct from actin filaments, intermediate filaments (IFs), and microtubules. BFs have a 6–8‐nm filament backbone upon which 12–15‐nm beads are distributed [1, 2, 3, 4, 5]. BFs consist of the lens fiber cell‐specific proteins, filensin (also called beaded filament structural protein 1 [BFSP1]; cytoskeletal protein 94 or 115 [CP94 or CP115], based on apparent molecular weight on SDS/PAGE) and phakinin (beaded filament structural protein 2 [BFSP2]; cytoskeletal protein 49 [CP49]) [3, 4, 5, 6, 7, 8, 9, 10, 11, 12, 13, 14, 15, 16, 17, 18, 19, 20]. Gene structure and primary amino acid sequence analyses show filensin and phakinin to be members of the IF protein family; however, the two proteins have several unique sequence characteristics that are not present in other IF proteins [3, 4, 5, 15, 17, 18, 19, 20, 21, 22]. Filensin consists of a tripartite structure that includes an N‐terminal head domain, a central α‐helical rod domain that can be divided into three subdomains (coil 1A, coil 1B, and coil 2), and a C‐terminal tail domain, which is similar to other IF proteins [23, 24, 25]. However, the rod domain of filensin is shorter than that in other IF proteins because of a 29 amino acid truncation within coil 2. IF proteins generally harbor the conserved helix initiation motif (HIM) sequence, that is, LNDR, at the N‐terminal end of the rod domain and the helix termination motif (HTM) sequence, that is, TYRKLLEGEE, at the C‐terminal end of the rod domain [23, 24, 25]. These two motifs are critical for IF assembly, and variations in these sequences can be disease‐causing [23, 26, 27, 28]. The HIM and HTM motif sequences in filensin are modified to LGER and RYHRIIE(I/N)EG, respectively [3, 4]. In addition, bovine filensin (CP115) includes a large extension in its tail domain that is absent from mouse and rat filensins (CP94), and chick filensin (CP95) [8, 9, 20, 22]. Phakinin consists of a head domain and an α‐helical rod domain but lacks a C‐terminal tail domain, except for trout phakinin [29]. The HTM sequence of phakinin is modified to SYHALLDREE, whereas the HIM sequence shows sequence divergence between vertebrate species [3, 4]. Filensin and phakinin, either purified from lenses or expressed recombinantly, coassemble to form IFs in cell‐free conditions, but the individual proteins cannot form IFs on their own [1, 11, 15, 16, 30, 31]. It has been proposed that filensin and phakinin form filaments by an assembly pathway that is distinct from those of other IF proteins [30]. It has also been proposed that the bead structures of native BFs are formed either intrinsically by filensin's long tail domain or extrinsically by α‐crystallins associated along the filensin/phakinin co‐polymerized filaments [16, 30]. When filensin and phakinin are co‐transfected into non‐lenticular cells, they form heteropolymeric filamentous networks in the cytoplasm, although expression of the individual proteins gives only aggregates [30, 31]. Therefore, the assembly experiments in cell‐free conditions and in cells clearly demonstrate that filensin and phakinin are required to partner each other to form filaments.

Beaded filaments are found exclusively in the fiber cells of the lens; therefore, it is assumed that they play unique functional roles in lens fiber cell biology. Deletion of filensin or phakinin in mice by gene targeting has revealed that absence of either protein results in a total absence of BFs, confirming that both are critical to BF assembly [32, 33, 34]. Although the lens fiber cells in these knockout mice appeared to differentiate normally, subtle loss of optical properties was identified, such as light scattering that grew worse with age and decay in the optical quality of the lens. Furthermore, structural changes in fiber cell shape and plasma membrane organization were observed in phakinin knockout mice [35, 36, 37]. BFs influence the mechanical properties and the geometry of lens fiber cells [38, 39].

Some forms of hereditary cataract in humans are caused by mutations of the filensin or phakinin genes. Sequence analysis of the phakinin gene from individuals with autosomal‐dominant congenital cataract showed an in‐frame deletion of a glutamic acid residue (∆E233) located in coil 1B of the rod domain [40, 41, 42, 43]. A missense mutation causing an R287W substitution in coil 2 of phakinin has been identified in a family with an autosomal‐dominant, juvenile‐onset, and progressive cataract [44]. Another missense mutation causing R339H in coil 2 of phakinin has been identified in a family with an autosomal‐dominant lamellar cataract [45], while an autosomal recessive, juvenile‐onset cataract was linked to a human genomic filensin sequence that lacks a 3.3‐kb sequence including exon 6 [46]. This mutation results in a protein that has the initial 245 wild‐type amino acid residues followed by six additional new residues and then a premature stop codon because of a frameshift. The protein therefore lacks the final 414 amino acids that form part of coil2 and the entire tail domain. It is not known whether the mRNA produced by the mutation is transcribed, or removed by the nonsense‐mediated decay RNA surveillance pathway, which would result in a filensin null allele. A missense mutation causing a D348N substitution in the tail domain of filensin was identified in a family with autosomal‐dominant congenital cataract [47]. Recently, a filensin frameshift variant (c.1124delA) that causes an E375G substitution and deletion of most of the tail domain was observed in a family with autosomal‐dominant pediatric cataract [48].

Beaded filament proteins undergo a reduction in molecular mass with fiber cell maturation. Phakinin (49 kDa) is proteolytically processed to a 40 kDa fragment [49]. Bovine filensin (115 kDa) is mainly divided into two fragments, an N‐terminal fragment including the N‐terminal head/central rod domains (53 kDa) and a C‐terminal fragment containing the tail domain (51–62 kDa) [8, 11, 50, 51, 52, 53]. The major proteolytic cleavage site of bovine filensin is D431 [54]. In concert with proteolytic processing, the subcellular distribution of filensin and phakinin in lens fiber cells changes as follows [8, 51, 53, 55, 56]. While intact filensin and phakinin are primarily located at the plasma membrane in peripheral lens fiber cells, the processed N‐terminal fragments of filensin and phakinin localize at the plasma membrane and in the cytoplasmic space in mature lens fiber cells. In contrast, the C‐terminal fragment of filensin remains associated with the plasma membrane of mature lens fiber cells. Recently, the C‐terminal fragment of filensin was identified to bind to aquaporin 0, which is the most abundant membrane protein in the lens and is assumed to play an important role in maintaining lens transparency and homeostasis [57, 58, 59, 60, 61, 62].

We have previously reported that in the Shumiya cataract rat (SCR) lens, filensin is excessively processed during cataractogenesis [56]. The SCR is a hereditary cataract rat model derived from a congenic line of SHR‐fa rats and 66.7% of the animals develop cataracts [63]. Lens opacity first appears in the nuclear and perinuclear regions in 11‐week‐old SCRs, and later develops into a mature cataract. In normal rat lens, phakinin is partially processed from the intact 49 kDa protein to a 40 kDa fragment. Filensin is processed to a certain extent from the intact 94 kDa protein into an N‐terminal 50 kDa fragment containing the central rod domain and a C‐terminal 36 kDa fragment containing the tail domain [56], similar to the processing of bovine filensin in normal lenses [22, 51, 52]. In the SCR lens, however, abundance of intact filensin and the N‐terminal 50 kDa fragment decrease significantly and the 38 kDa fragment becomes the main fragment before opacification [56]. Amino acid sequence analysis of the filensin fragments indicated that the N‐terminal 29 amino acids are deleted [64]. Immunohistochemical studies using an anti‐phakinin antibody and an anti‐filensin rod domain antibody revealed that in precataract lenses, signals of both phakinin and filensin localize primarily to the membranes lining the lens fiber cells in the shallow cortex and that they are also distributed throughout the cytoplasm of the lens fiber cells in the deep cortex. This is in contrast with signals in the deep cortex of the normal rat lens, where they localize to the central region of the cytoplasm [56, 65]. It is unknown whether the excessive degradation into the 38 kDa fragment affects filensin function and the BF structures in the rat lens fiber cells.

In this study, we prepared recombinant rat filensin fragments representing the 38 and 50 kDa fragments, and compared their competencies to form heteropolymeric filaments with recombinant rat phakinin in cell‐free conditions. We also co‐transfected SW‐13 and MCF‐7 cells with genes encoding filensin fragments and phakinin that were fused with fluorescent proteins, and examined filament formation in the cells. A filensin fragment, Fil(30–416) containing residues 30–416, and another fragment, Fil(30–369) containing residues 30–369 of rat filensin (664 amino acids), were employed to represent the 50 and 38 kDa fragments, respectively. Fil(30–416) and full‐length filensin formed heteropolymeric filaments with phakinin, while Fil(30–369) did not form filaments with phakinin and produced aggregates both in cell‐free conditions and in cells. These results indicate that the rat filensin tail B region, residues 370–416, is essential for filament formation and that truncation in this region results in loss of function. Deletion of the N‐terminal 29 amino acids, which form most of the filensin head domain, did not affect filament formation. These results give insight into the mechanisms underlying cataractogenesis by the excessive proteolysis of filensin in lens fiber cells.

## Materials and methods

### Plasmid construction and expression of recombinant proteins in *Escherichia coli* cells

A cDNA (2 kb) encoding rat filensin (abbreviated as Fil, accession number: XM_342529, 664 amino acids) and a cDNA (1.3 kb) encoding rat phakinin (abbreviated as Phk, accession number: XM_001069678, 416 amino acids) were generated from rat lens total RNA, cloned into appropriate plasmids, including pT7Blue (Novagen, Madison, WI, USA), and then confirmed by sequencing, as described previously [56, 66]. For expression in *E. coli* cells, the filensin and phakinin genes were subcloned into pET24c (Novagen) and pCold IIII (Takara Bio USA, Mountain View, CA, USA) vectors, respectively, using NdeI and HindIII cloning sites. A gene encoding a filensin fragment lacking the N‐terminal 29 and C‐terminal 248 amino acids, Fil(30–416), was obtained by PCR, using the filensin cDNA as a template, a forward primer FilNdeI‐New (5′‐AACCCCATATGACCGCGCCCGGCCTG‐3′) and a reverse primer Fil50HindIII‐long (5′‐GGACCAAGCTTTCATGTTGGACTTACTTCA‐3′). Similarly, a gene encoding the other filensin fragment lacking the N‐terminal 29 and C‐terminal 295 amino acids, Fil(30–369), was obtained by PCR, using the filensin cDNA as a template, the forward primer FilNdeI‐New and a reverse primer Fil38HindIII‐long (5′‐TTATCAAGCTTTCAAATCTCCTTTCTTTTT‐3′). The genes encoding the filensin fragments were inserted into expression vector pET28b (Novagen) using NdeI and HindIII cloning sites and confirmed by DNA sequencing. The genes encoding filensin, Fil(30–416) and Fil(30–369), were expressed in *E. coli* strain Rosetta(DE3)pLysS (Novagen) using isopropyl‐β‐d‐thiogalactopyranoside (IPTG) induction. The gene encoding phakinin was expressed in Rosetta2 (Novagen) using IPTG and cold‐shock induction. The recombinant proteins were enriched in the inclusion body, which was isolated by the method of Nagai and Thogersen [67], extracted with 8 m urea, and then purified by anion exchange column chromatography in the presence of 6 m urea, as described [68]. The purified proteins were dialyzed against buffer A containing 10 mm Tris–HCl, pH 8.0, 8.5 m urea, 1 mm EDTA, 10 mm DTT, 0.2 mm PMSF, and protease inhibitor cocktail, cleared of aggregates by centrifugation at 100 000 ***g*** at 10 °C for 1 h, and stored at −80 °C. Protein concentration was determined by the method of Bradford [69].

### Cell‐free assembly

Filensin and phakinin in buffer A were mixed at a molecular ratio of 1 : 2 and at a total concentration of 0.1–0.5 mg·mL^−1^, and dialyzed against buffer B containing 20 mm Tris–HCl, pH 7.4, 8.5 m urea, 1 mm MgCl_2_, 0.1 mm EGTA, and 10 mm DTT at 25 °C overnight, then against buffer C containing 20 mm Tris–HCl, pH 7.4, 50–100 mm KCl, 1 mm MgCl_2_, 0.1 mm EGTA, and 10 mm DTT at 25 °C for 3 h. Dialysis against buffer C was carried out two more times after changing buffer C. In these procedures, all buffers were degassed and then stored under N_2_ before use, and the dialysis was carried out in anaerobic bags filled with N_2_ gas.

### Electron microscopy

For negative staining, a 20 μL aliquot of each sample was adsorbed onto a collodion film on a copper grid, and then stained with 2% uranyl acetate as described [68]. Samples were examined using a JEM‐1210 transmission electron microscope (JEOL, Tokyo, Japan) that was operated at an accelerating voltage of 80 kV.

### Plasmid construction for expression in cultured cells

To subclone the filensin gene into the expression vector, pAcGFP1‐Hyg‐N1 (Takara Bio USA), the filensin cDNA was amplified by PCR using a forward primer Fil94‐5′‐XhoI‐long (5′‐TATAAACTCGAGCCACCATGTACCGCCGCAGCTACGTCTT‐3′) containing an XhoI site and the Kozak sequence (both underlined) followed by the initiation codon, and a reverse primer Fil94‐3′‐HindIII (5′‐GGCCGCAAGCTTTCCAGCCTTGGCATTTGAGGA‐3′) containing a HindIII site and TCC corresponding to a Gly residue (both underlined) in place of the stop codon. The resultant gene was purified, treated with the restriction enzymes, and ligated into pAcGFP1‐Hyg‐N1 (Takara Bio USA). The resultant plasmid, pAcGFP1‐Fil, was confirmed by sequencing. This plasmid expresses the filensin sequence followed by a linker sequence of Gly‐Lys‐Leu‐Arg‐Ile‐Leu‐Gln‐Ser‐Thr‐Val‐Pro‐Arg‐Ala‐Arg‐Asp‐Pro‐Pro‐Val and the *Aequorea coerulescens* green fluorescent protein (AcGFP1) sequence. Similarly, the phakinin gene was amplified by PCR using a forward primer Phk‐5′‐XhoI (5′‐ATGAATCTCGAGGCCACCATGAGCGAGAGGAGAGT‐3′) containing an XhoI site and the Kozak sequence (both underlined) followed by the initiation codon, and a reverse primer Phk‐3‐HindIII (5′‐GGTCGACAAGCTTTCCGTTGCTCTCCTCTCTGTC‐3′) containing a HindIII site and TCC corresponding to a Gly residue (both underlined) in place of the stop codon. The resultant gene was purified, treated with the restriction enzymes, and ligated into pmCherry‐N1 (Takara Bio USA). The resultant plasmid, pmCherry‐Phk, was confirmed by sequencing. This plasmid expresses the phakinin sequence followed by a linker sequence of Gly‐Lys‐Leu‐Arg‐Ile‐Leu‐Gln‐Ser‐Thr‐Val‐Pro‐Arg‐Ala‐Arg‐Asp‐Pro‐Pro‐Val‐Ala‐Thr and the monomeric Cherry fluorescent protein (mCherry) sequence.

The gene coding the filensin fragment, Fil(30–416), was also prepared by PCR, using the rat filensin cDNA as a template, the forward primer Fil38‐XhoI‐long (5′‐ATTATTTTACTCGAGGCCACCATGACCGCGCCCGGCCTGGCGGCGCTGCAGGCACTG‐3′) containing an XhoI site and the Kozak sequence (both underlined) followed by the initiation codon, and a reverse primer Fil50‐HindIII (5′‐TCAGGACCAAGCTTTCCTGTTGGACTTACTTCATGTCC‐3′) containing a HindIII site and TCC corresponding to a Gly residue (both underlined) in place of the stop codon. The resultant genes were treated with XhoI and HindIII, and ligated into pAcGFP1‐Hyg‐N1 (Takara Bio USA). The gene coding the other filensin fragment, Fil(30–369), was prepared by PCR using the rat filensin cDNA as a template, the forward primer Fil38‐XhoI‐long, and a reverse primer Fil38‐HindIII (5′‐GCGGCCGCAAGCTTTCCAATCTCCTTTCTTTTTGGAAG‐3′) containing a HindIII site and TCC corresponding to a Gly residue (both underlined) in place of the stop codon. The resultant plasmids pAcGFP1‐Fil(30–416) and pAcGFP1‐Fil(30–369) were confirmed by sequencing. These plasmids express the filensin fragments, Fil(30–416) and Fil(30–369), respectively, followed by the linker sequence and the AcGFP1 protein. Prior to use for transfection, all constructs, pAcGFP1‐Fil, pAcGFP1‐Fil(30–369), pAcGFP1‐Fil(30–416), and pmCherry‐Phk, were purified using an EndoFree Plasmid Maxi Kit (Qiagen, Hilden, Germany).

### Cell culture and transfection

MCF‐7 cells (a human mammary carcinoma cell line) and SW‐13 cells (a human adenocarcinoma cell line derived from adrenal cortex) were purchased from the Japanese Collection of Research Bioresources Cell Bank. The cells were grown in Dulbecco's modified Eagle's medium/Ham's F‐12 medium (Fujifilm Wako Pure Chemical, Osaka, Japan) supplemented with 10% fetal calf serum, in a 5% CO_2_ atmosphere at 37 °C. SW‐13 cells expressing only vimentin as an endogenous IF protein were cloned, confirmed by SDS/PAGE and western blotting, and used for subsequent experiments as described [70]. Transfection of MCF‐7 and SW‐13 cells with plasmids was performed by lipofection using ScreenFect A Plus (Fujifilm Wako Pure Chemical), according to the manufacturer's protocol. Briefly, 1 × 10^5^ SW‐13 cells or 6 × 10^5^ MCF‐7 cells were placed onto a sterile 18 × 18 mm coverslip in a 35 mm culture dish and cultured in 2 mL of the above medium for 3 days. When cell density reached 70–90% confluency (i.e., approximately 1 × 10^6^ cells in a dish), the medium was replaced with fresh medium, and transfection cocktail (240 μL) was added. The cells were then incubated for 2 further days. The transfection cocktail was prepared by mixing the filensin (or filensin fragment) and phakinin plasmid DNAs at a molar ratio of 1 : 2, respectively (total amount of plasmid DNAs was adjusted at 1.25 or 2.50 μg), ScreenFect A Plus (four times the amount of the DNA mixture, that is, 5 or 10 μg), and transfection reagent dilution buffer.

### Immunocytochemistry

Cells grown on glass coverslips were fixed in 4% formaldehyde in PBS at room temperature for 15 min and permeabilized with 0.1% Triton X‐100 in PBS at room temperature for 5 min. To detect endogenous vimentin in SW‐13 cells, the cells were incubated overnight at 4 °C with rabbit anti‐vimentin IgG (1 : 100 dilution; Cell Signaling Technology, Danvers, MA, USA). After washing three times with PBS, the cells were incubated for 1 h with CF350‐conjugated goat anti‐rabbit IgG antibody (1 : 200 dilution; Biotium, Fremont, CA, USA). When required, cell nuclei were stained with 1 μg·mL^−1^ 4′,6‐diamidino‐2‐phenylindole (DAPI; Dojindo, Kumamoto, Japan) for 5 min at room temperature. Fluorescently labeled cells were examined using an Olympus BX53 fluorescence microscope (Olympus, Tokyo, Japan).

## Results and Discussion

### Preparation of recombinant proteins

**Fig. 1:**
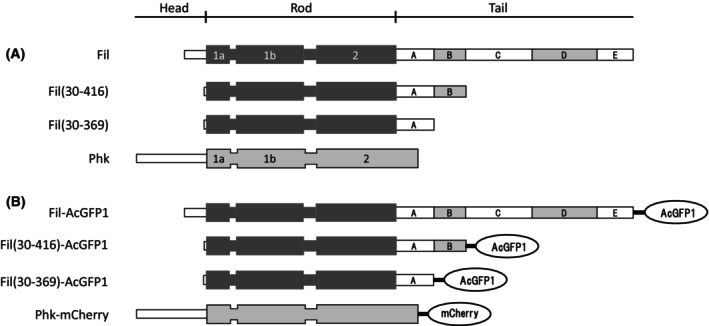
Schematic representation of the structural domains of rat filensin, filensin fragments, and phakinin. (A) Rat filensin (Fil) consists of the N‐terminal head domain (33 amino acids), the central rod domain (280 amino acids) containing α‐helical coils 1a, 1b, and 2, and the C‐terminal tail domain (351 amino acids). The tail domain is composed of three conserved regions (tail A, C, and E), which are interrupted by two regions (tail B and D) with lower identity [22]. The filensin fragment Fil(30–416) lacks the N‐terminal 29 amino acids in the head domain and the C‐terminal 248 amino acids corresponding to the tail C–E regions. The filensin fragment Fil(30–369) lacks the N‐terminal 29 amino acids in the head domain and the C‐terminal 295 amino acids corresponding to the tail B–E regions. Rat phakinin consists of the N‐terminal head domain (105 amino acids) and the rod domain (311 amino acids). (B) For expression in cultured cells, filensin, Fil(30–416), and Fil(30–369), were C‐terminally tagged with AcGFP1 fluorescent protein. Phakinin was C‐terminally tagged with mCherry fluorescent protein.

In this study, recombinant rat filensin, phakinin, and two kinds of filensin fragments, Fil(30–416) and Fil(30–369) (Fig. 1A), were prepared using *E. coli* expression systems. Fil(30–416), lacking the N‐terminal 29 amino acids and the C‐terminal 248 amino acids of rat filensin (664 amino acids), has a theoretical molecular weight of 43 416 and mimics the 50 kDa filensin fragment observed in rat lens fiber cells [56]. Another fragment, Fil(30–369), lacking the N‐terminal 29 amino acids and the C‐terminal 295 amino acids of rat filensin, has a theoretical molecular weight of 38 631 and mimics the 38 kDa filensin fragment observed in SCR lens fiber cells [56]. It has been proposed that the filensin tail domain is composed of three conserved regions (tail A, C, and E), which are interrupted by two regions (tail B and D) with lower identity [22]. In rat filensin, the tail regions correspond to the following residues; tail A: residues 314–369, tail B: 370–416, tail C: 417–515, tail D: 516–611, and tail E: 612–664 [22]. Therefore, Fil(30–416) has the tail A and B regions, but lacks the tail C–E regions, whereas Fil(30–369) only has the tail A region.

The rise in calcium concentrations with age is more marked in the SCR lens compared with that in normal rat lens. This leads to activation of calpains (calcium‐dependent cysteine proteases), which causes the proteolytic processing of lens proteins, including α‐ and β‐crystallins [71]. Bovine filensin is also susceptible to degradation by calpains [72]. We previously showed that both the 38 and 50 kDa filensin fragments observed in the SCR lens lack the N‐terminal 29 amino acid residues, indicating cleavage of the G^29^‐T^30^ peptide bond in the sequence ‐P‐A‐G‐P‐T‐A‐Q‐P^27^‐G‐G^29^‐T^30^‐A‐P^32^‐G‐L‐A‐A‐ (the head domain sequence is underlined) [56, 64]. This cleavage site conforms with the substrate recognition sequence of calpains because calpains preferentially cleave peptide bonds (P_1_‐P_1′_) surrounded by proline residues, especially with a proline at the P_3′_ position [73, 74, 75]. However, the C‐terminal residues of the 38 and 50 kDa filensin fragments remain unknown. Our previous western blotting analysis using two antisera that were raised against recombinant peptides representing the rat filensin sequences from coil 1b to tail A and from tail B to tail C showed that while the 50 kDa filensin fragment harbors both tail A and B regions, the 38 kDa filensin fragment contains only the tail A but not the tail B region [56]. In the relatively conserved C‐terminal region of the tail A sequence, ‐T^350^‐A‐A‐K‐ P^354^‐R‐Q‐K‐A‐L‐P^360^‐K‐S‐L‐P^364^‐K‐R‐K‐E^368^‐I^369^ ‐I‐A‐Q‐ (the tail A region is underlined), three proline residues, P^354^, P^360^, and P^364^ might act as a substrate recognition determinant for calpains. Using this information and an apparent molecular weight observed in SDS/PAGE analysis [56], we employed Fil(30–369) to mimic the 38 kDa filensin fragment, although a shorter tail A sequence than that of Fil(30–369) might arise following truncation by calpains. We estimated that the C‐terminus of the 50 kDa filensin fragment would be a residue located on the N‐terminal side of residue D^426^ in the boundary sequence between the tail B and C regions, G^410^‐H‐E‐V‐S‐P^415^‐T^416^ ‐Q‐E‐G‐G‐P^421^‐E‐D‐V‐P^425^‐D^426^‐G^427^‐S‐Q‐I‐S‐K‐A‐F^434^‐ (the tail B sequence is underlined; the conserved sequence in tail C is double underlined). The D^426^ residue corresponds to D^431^ in bovine filensin and also to D^433^ in human filensin, both of which are the major cleavage sites for caspases to form the 53 kDa fragments during differentiation and aging of lens fiber cells [22, 50, 51, 54, 59]. In the boundary sequence between the tail B and C regions, the three proline residues, P^415^, P^421^, and P^425^, might act as a substrate recognition determinant for calpains. Using this information and an apparent molecular weight observed in SDS/PAGE analysis [56], we employed Fil(30–416) to mimic the 50 kDa filensin fragment. Therefore, although Fil(30–369) and Fil(30–416) might not have the same C‐termini as the 38 and 50 kDa filensin fragments observed in the SCR lens, the two recombinant fragments were expected to be useful for understanding the effects of truncations in the terminal domain structures on the heteropolymeric filament formation with phakinin.

**Fig. 2:**
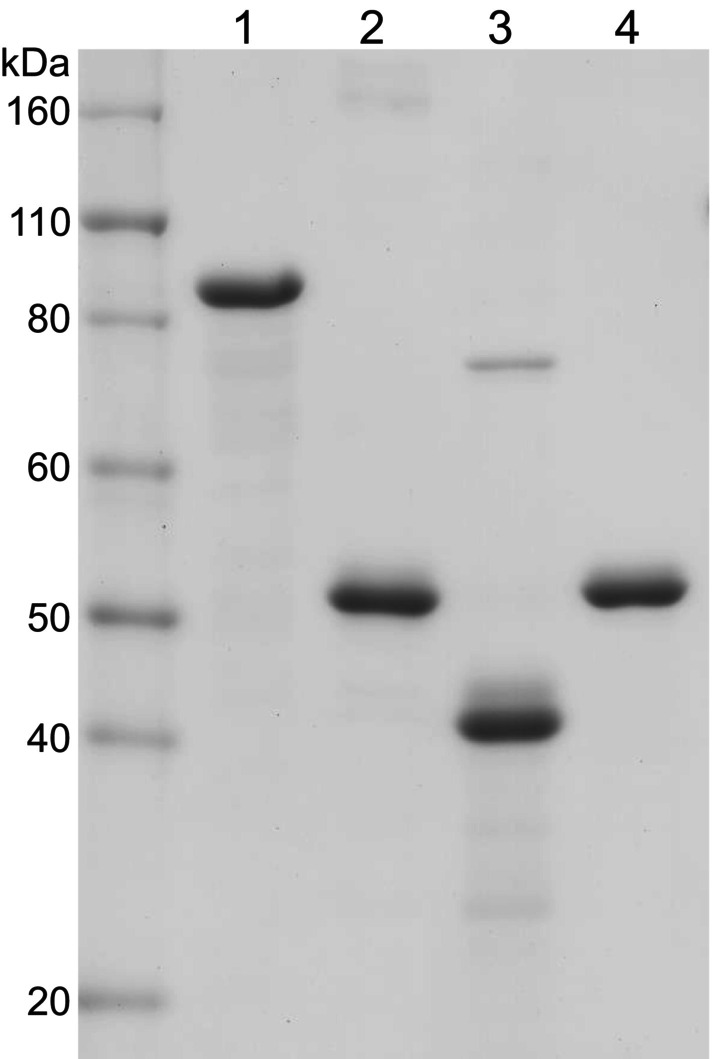
SDS/PAGE of recombinant rat filensin, filensin fragments, and phakinin prepared in this study. The purified polypeptides were resolved on 7.5% polyacrylamide gels and stained with Coomassie brilliant blue: lane 1, filensin; lane 2, Fil(30–416); lane 3, Fil(30–369); lane 4, phakinin. Protein size (kDa) is indicated on the left.

Both recombinant filensin fragments as well as recombinant filensin and phakinin were generated in inclusion bodies and purified by conventional ion‐exchange column chromatography in the presence of 6 m urea. Slight differences between theoretical and apparent molecular weights of filensin and the filensin fragments on SDS/PAGE (Fig. 2) are likely related to the anomalous behavior of bovine filensin on SDS/PAGE; it behaves as having a higher molecular weight of 115 kDa compared with its theoretical molecular weight of 83 kDa [54]. Although the precise reason for this remains unknown, the tail domain sequence is likely to participate in the anomaly [54].

### Cell‐free assembly of recombinant filensin fragments and phakinin

**Fig. 3:**
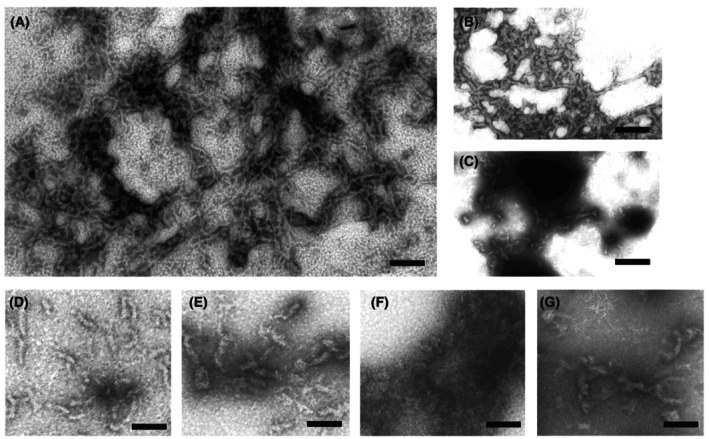
Electron microscopy of products assembled in cell‐free conditions. Combinations of (A) filensin/phakinin, (B) Fil(30–416)/phakinin, (C) Fil(30–369)/phakinin, and individual (D) filensin, (E) Fil(30–416), (F) Fil(30–369), and (G) phakinin were assembled as described in the ‘Materials and methods’ section. The samples were contrasted by negative staining. Scale bars, 200 nm (A–C) and 50 nm (D–G).

Recombinant rat filensin or filensin fragments were mixed with recombinant phakinin at a molecular ratio of 1 : 2 in the presence of 8.5 m urea, consistent with the reported molar ratio of the proteins that were extracted from bovine lenses [15, 16]. Cell‐free assembly was performed by dialysis under an N_2_ atmosphere to remove urea and adjust the pH and ionic strength, with reference to the methods reported for bovine filensin and phakinin [15, 16, 30, 31]. As shown in Fig. 3A,B, the pairs of filensin/phakinin and Fil(30–416)/phakinin formed heteropolymeric filaments. The filaments observed were relatively rugged and entangled when compared with filaments observed for bovine filensin and phakinin [15, 16, 30]. The bead structures characteristic for native BFs were not observed. In contrast, the Fil(30–369)/phakinin pair gave only aggregates (Fig. 3C). Under the same assembly conditions, individual filensin and Fil(30–416) gave short rod‐like structures (Fig. 3D,E), as observed for bovine filensin [15, 16, 30] and porcine filensin [11], whereas Fil(30–369) gave only aggregates (Fig. 3F). Recombinant rat phakinin self‐assembled into short fibrillar or globular structures (Fig. 3G), as reported for bovine phakinin [10]. Therefore, Fil(30–369) lacking the B–E tail regions was unable to form filaments with phakinin or to self‐assemble into short rod‐like structures. Interestingly, truncation of the N‐terminal 29 amino acids covering most of the 33 amino acid head domain of filensin did not interfere with filament formation with phakinin, although the head domains of authentic IF proteins are indispensable for IF formation [23, 24, 25, 76].

### Expression of filensin fragments and phakinin in cultured cells

To characterize further the assembly properties of the filensin fragments, Fil(30–369) and Fil(30–416), they were transiently co‐expressed with phakinin in cultured cells. The filensin fragments and filensin were expressed as fusion proteins with the green fluorescent protein, AcGFP1, and phakinin was expressed as a fusion protein with the red fluorescent protein, mCherry (Fig. 1B). Fluorescent proteins fused to the C terminus of IF proteins do not interfere with IF formation and facilitate detection of the structures and dynamics of IFs in cells using fluorescence microscopy [70, 77, 78]. For transient expression of the filensin fragments and phakinin, we selected SW‐13 and MCF‐7 cells. MCF‐7 cells are simple epithelial cells of human mammary carcinoma origin, and endogenously express cytokeratin 8, 18, and 19 (type I and II IF proteins) [79, 80]. Exogenously expressed filensin and phakinin do not co‐polymerize with endogenous keratins [30]. SW‐13 cells are derived from a non‐epithelial human adrenal cortex carcinoma and lack endogenous IF proteins [81, 82], although a few SW‐13 cells do express vimentin (a type III IF protein) [82]. We have previously cloned vimentin‐expressing SW‐13 cells [70] and used them to provide endogenous vimentin IFs as a reference for cytoplasmic IF arrays. The filensin fragment or filensin gene was mixed with the phakinin gene at a molar ratio of 1 : 2 and then transfected into cultured SW‐13 or MCF‐7 cells by lipofection [83].

Transfection experiments of each protein pair (filensin/phakinin, Fil(30–416)/phakinin or Fil(30–369)/phakinin) were performed at least three times for SW‐13 cells and MCF‐7 cells. Approximately 80% of the transfected cells expressed both filensin (or filensin fragments) and phakinin. The remaining transfected cells expressed only phakinin, as observed by fluorescence microscopy (Figs S1 and S2).

**Fig. 4:**
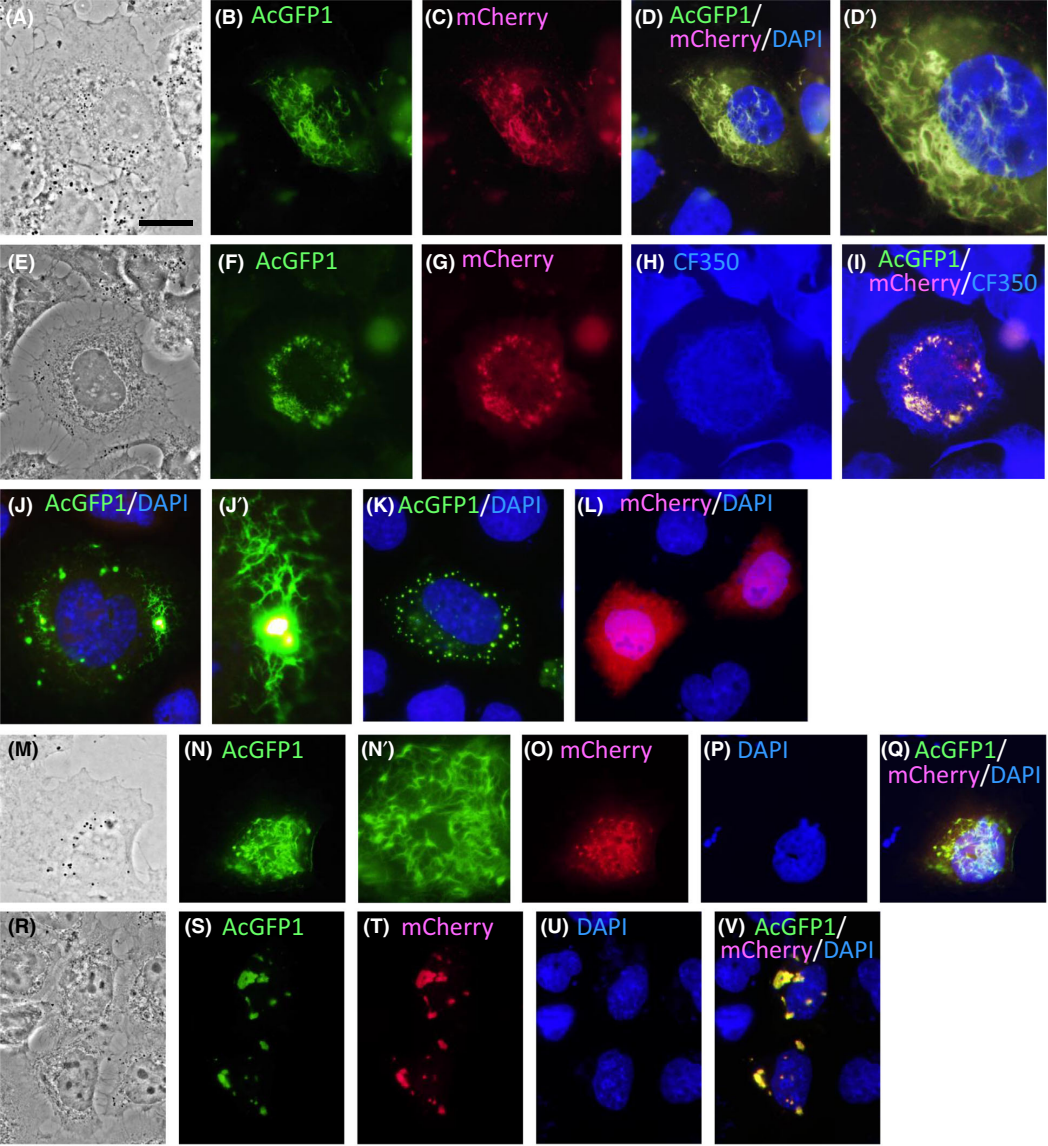
Expression of fluorescent protein‐tagged rat filensin, filensin fragments, and phakinin in SW‐13 cells. Pairs of filensin/phakinin (A–J′), Fil(30–416)/phakinin (M–Q), and Fil(30–369)/phakinin (R–V), and individual filensin (K) and phakinin (L) were transiently expressed in SW‐13 cells. Phase contrast images (A, E, M, R). Fluorescence images of the filensin proteins tagged with AcGFP1 [B: filensin‐AcGFP1, F: filensin‐AcGFP1, J and J′: filensin‐AcGFP1, K: filensin‐AcGFP1, N and N′: Fil(30–416)‐AcGFP1, S: Fil(30–369)‐AcGFP1], phakinin tagged with mCherry (C, G, L, O, T), endogenous vimentin immunologically visualized with CF350 (H) and nuclei stained with DAPI (D, D′, J, K, L, P, U) were observed and merged (D, D′, I, J, J′, K, L, Q, V). Fluorescence images of (D), (J), and (N) are enlarged in (D′), (D′), and (N′), respectively. Note that the pairs of filensin/phakinin (A–J′) and Fil(30–416)/phakinin (M–Q) formed sinuous and mesh‐like filaments (B–D′, J, J′, and N–Q) or many foci from which short filaments extrude radially (F–I). In contrast, the Fil(30–369)/phakinin pair (R–V) gave aggregates (S–V). Scale bars: 20 μm.

In SW‐13 cells, authentic cytoplasmic IFs form filament networks spreading throughout the cytoplasmic space [84, 85, 86]; however, the exogenously expressed rat filensin/phakinin pair formed cytoplasmic filaments that were intrinsically different. The rat filensin/phakinin filaments were sinuous and had different widths (Fig. 4A–D′,J,J′; Fig. S1A). Interestingly, some filaments appeared to be mesh‐like by branching and joining each other (Fig. 4D′,J′). In other specimens, the filensin/phakinin pair formed many foci from which short filaments extrude radially (Fig. 4E–G,I). These structures might reflect the intrinsic structural features of the BFs as observed by conical tomography of lens fiber cells, in which the BFs are composed of bent filaments that cross each other and are studded with particles [87]. In addition to the filaments, a few nonfilamentous (or spherical) aggregates were also occasionally observed in these cells, as shown in Fig. 4J,J′, Fig. S1A, and Table S1 (statistical analysis data shown). Therefore, the filensin/phakinin pair showed some different structural phenotypes in SW‐13 cells (Fig. 4A–J′; Fig. S1A), compared with authentic IF proteins that form long and regular filaments spreading throughout the cytoplasmic space [84, 85, 86]; however, the reason for this remains unknown. The filaments of rat filensin/phakinin did not co‐localize with endogenous vimentin filaments (Fig. 4F–I), indicating that filensin and phakinin co‐polymerize to form *de novo* filaments. These results support the view that the filensin/phakinin pair forms filament networks in cells that are distinct from vimentin filaments [88], but are inconsistent with the observation of the exogenously expressed bovine filensin/phakinin pair co‐localizing with endogenous vimentin filaments in CHO cells [30, 31]. When rat filensin or phakinin was individually expressed in SW‐13 cells, it formed only dot‐like aggregates or fine aggregates, respectively, in the cytoplasm (Fig. 4K,L), supporting the idea that both filensin and phakinin are necessary for filament formation in cells [30]. The Fil(30–416)/phakinin pair also formed sinuous, mesh‐like filaments of different widths in SW‐13 cells (Fig. 4M–Q; Fig. S1B), while some aggregates were also occasionally observed in these cells (Fig. S1B; Table S1). In contrast, the Fil(30–369)/phakinin pair produced mostly aggregates in the cytoplasmic space of SW‐13 cells (Fig. 4R–V; Fig. S1C; Table S1), although there remains a possibility that aggregates might be composed of fibrillar elements that easily tangle with each other.

**Fig. 5:**
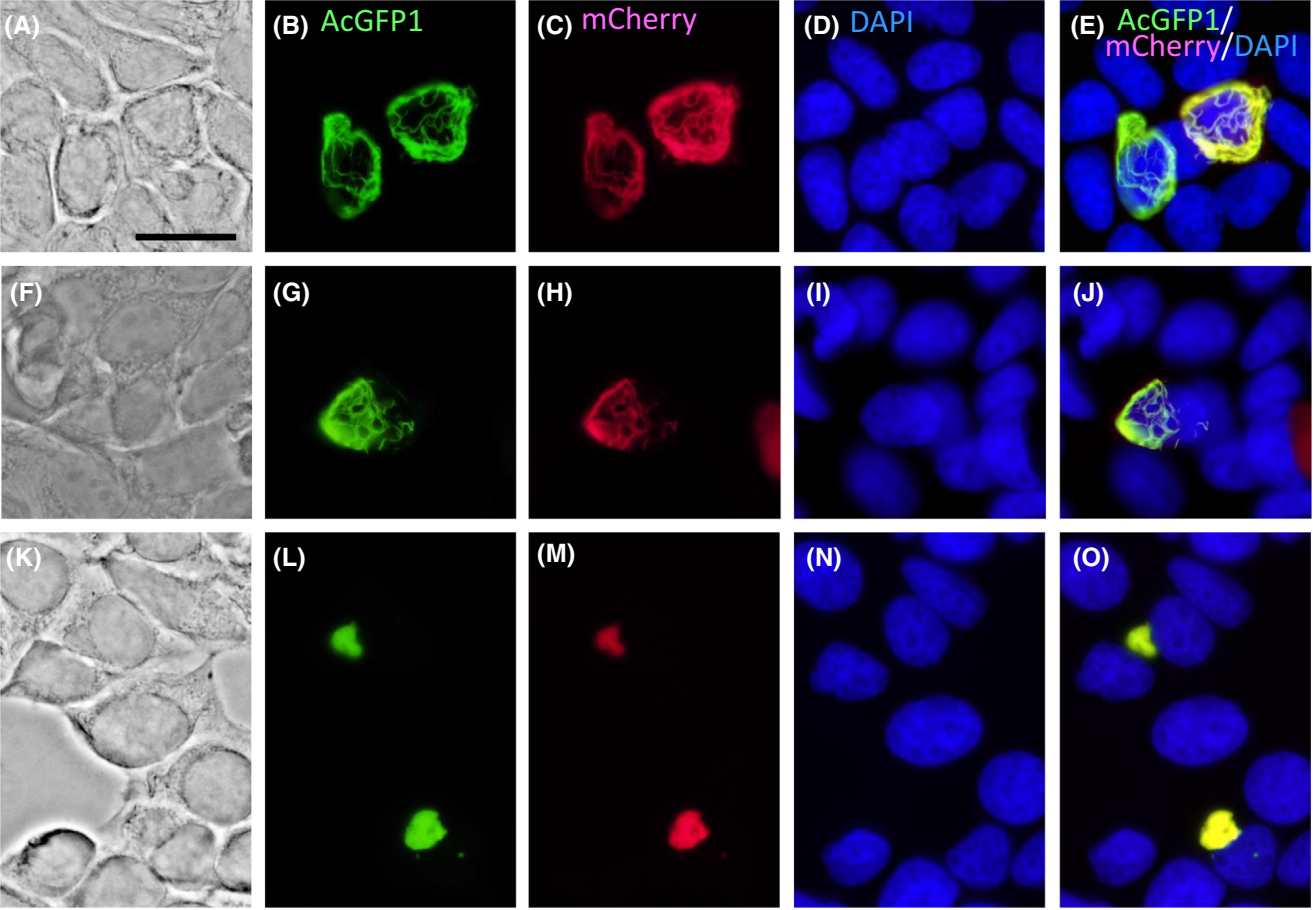
Expression of fluorescent protein‐tagged rat filensin, filensin fragments, and phakinin in MCF‐7 cells. Pairs of filensin/phakinin (A–E), Fil(30–416)/phakinin (F–J), and Fil(30–369)/phakinin (K–O) were transiently expressed in MCF‐7 cells. Phase contrast images (A, F, K). Fluorescence images of the filensin proteins tagged with AcGFP1 (B: filensin‐AcGFP1, G: Fil(30–416)‐AcGFP1, L: Fil(30–369)‐AcGFP1), phakinin tagged with mCherry (C, H, M), and nuclei stained with DAPI (D, I, N) were observed and merged (E, J, O). Note that the pairs of filensin/phakinin (A–E) and Fil(30–416)/phakinin (F–J) formed filaments that were sinuous and had different widths (B, C, E, G, H, and J). In contrast, the Fil(30–369)/phakinin pair (K–O) yielded aggregates (L, M, O). Scale bars: 20 μm.

In MCF‐7 cells, the rat filensin/phakinin and Fil(30–416)/phakinin pairs formed cytoplasmic filaments that were also sinuous and had different widths (Fig. 5A–J; Fig. S2A,B). Although it might be related to smaller cytoplasmic spaces of MCF‐7 cells compared with those of SW‐13 cells, the filaments appeared to be thick along the cell periphery. In MCF‐7 cells, a bovine filensin/phakinin pair forms distinct foci, from which filaments radiate toward the cytoplasm [30]; however, similar structures were not observed in this study. In contrast to the above two pairs, the Fil(30–369)/phakinin pair yielded many aggregates (Fig. 5K–O; Fig. S2C), indicating that deletion of the tail B region resulted in loss of the ability to form filaments.

Fil(30–416), lacking both the N‐terminal 29 residues in the head domain and the tail C–E regions, co‐polymerized with phakinin and formed filaments in cell‐free conditions and in cultured cells. Truncation of the N‐terminal 29 amino acids of rat filensin, covering most of the 33 amino acid head domain, did not interfere with the heteropolymeric filament formation with phakinin, although the head domains of authentic IF proteins have been shown to be indispensable for filament formation [23, 24, 25, 76]. Dispensability of the N‐terminal 29 residues of rat filensin for filament formation indicates a unique assembly mechanism for filensin and phakinin. While bovine filensin fragments lacking the whole tail domain do not form filaments with phakinin [31], the results obtained here indicate that tail A and B regions are sufficient for filament formation and that the tail C–E regions are not essential for filament formation. This result accounts for the fact that the bovine filensin 53 kDa fragments produced by proteolytic processing in the lens can form heteropolymeric filaments with phakinin [22, 89]. The proteolytic processing of bovine filensin occurs during lens fiber cell differentiation. Truncation at D431, probably by group II caspases, such as caspase 2, 3, and 7, yields the N‐terminal 53 kDa filensin fragment [54]. The truncation site D431 (corresponding to D426 for rat filensin and D433 for human filensin) is located close to the beginning of the tail C region; therefore, the resultant N‐terminal 53 kDa fragment likely contains the head/rod domains, the tail A–B regions and the N‐terminal 10 residues of the tail C region. Meanwhile, the C‐terminal ~ 50 kDa fragment covering the rest of the tail domain is subsequently myristoylated at the newly formed N terminus, binds to the membrane protein, aquaporin 0, and localizes to the cell membrane of lens fiber cells [51, 54, 56, 57, 58]. Aquaporin 0 is the most abundant membrane protein in the lens and plays an important role in maintaining lens transparency and homeostasis [60, 90]. Recently, a binding site for aquaporin 0 was identified in a conserved sequence of the tail C region of bovine filensin [62].

Fil(30–369) failed to form filaments with phakinin in cell‐free conditions and in cultured cells, indicating that the tail B region (residues 370–416) is indispensable for filament formation. This result accounts for a frameshift mutation in human filensin, p.E375GfsTer2 (c.1124delA), causing autosomal‐dominant cataract [48]. E375, corresponding to E368 in the rat filensin sequence (‐K‐R‐K‐E^368^‐I^369^ ‐I‐A‐Q‐: the tail A region is underlined), is located close to the C terminus of the tail A region; therefore, this frameshift mutation gives a filensin fragment that contains only the tail A region but not the rest of the tail domain. Truncation of the tail B region results in the loss of competence of filensin to form filaments. Therefore, the excessive proteolytic processing of rat filensin into the 38 kDa fragment observed in the SCR lens might interfere with filament formation and disturb the BF structure. However, the exact role of the tail B region in filament formation remains to be determined. The tail B region does not have a notable conserved amino acid sequence among species, although the region commonly has a very low pI value (pI 3–4) because of a relatively high acidic residue content (17–29%) and is rich in bulky residues, such as valine and leucine (13–21%). Prediction of a rat filensin 3D‐structure by AlphaFold2 (https://colab.research.google.com/github/sokrypton/ColabFold/blob/main/AlphaFold2.ipynb) does not show the tail B region taking on a specific structure. In contrast, the tail A region, having a conserved sequence and a high pI value (pI 10–11) because of a high content of basic residues, is predicted to have α‐helical structures for the six N‐terminal residues that extend the α‐helical structure running from the rod domain and for the central part of the tail A region, residues 340–350 (data not shown). The central part of the tail A region contains D341, which corresponds to the D348N mutation site of human autosomal‐dominant congenital cataract [47]. Since the tail domains of authentic IF proteins play a subtle role in IF formation, stabilization, and mechanical intensity [23, 24, 25], it is conceivable that the tail A and B regions of filensin are important for BF formation and stabilization.

In conclusion, our results indicate that the tail B region in rat filensin is essential to form heteropolymeric filaments with phakinin, whereas the N‐terminal head domain is not required to form filaments. These results also support the view that the tail domain of filensin has at least two distinct functional roles, filament formation with phakinin and interaction with aquaporin 0. Truncation of the tail B region to give the filensin 38 kDa fragment through excessive proteolytic processing in SCR lens fiber cells interferes with filensin's function in filament formation and disturbs the BF structure, thereby leading to opacification in the cataract rat lens. Further work is required to elucidate the role of the tail A and B regions in filament formation with phakinin.

## Conflict of interest

The authors declare no conflict of interest.

### Peer review

The peer review history for this article is available at https://www.webofscience.com/api/gateway/wos/peer‐review/10.1002/2211‐5463.13700.

## Author contributions

MoT and AN constructed the expression systems in cells and performed microscopy analysis. AN, MiT, and SI performed cell‐free assembly experiments. YK performed SDS/PAGE analysis and 3D‐structure prediction. MO cloned cDNAs from the rat lens and critically read the manuscript. SA conceived the project and wrote the manuscript.

## Ethics Statement

The cDNA cloning experiments of rat filensin and phakinin by M. O. conformed to the guidelines of the Committee of the Ethics of Animal Experiments at Keio University (Tokyo, Japan).

## Supporting information


**Fig. S1.** Expression of fluorescent protein‐tagged rat filensin, filensin fragments, and phakinin in SW‐13 cells. Pairs of filensin/phakinin (A), Fil(30–416)/phakinin (B) and Fil(30–369)/phakinin (C) were transiently expressed in SW‐13 cells. Fluorescence images of the filensin proteins tagged with AcGFP1 (green), phakinin tagged with mCherry (red), and nuclei stained with DAPI (blue) were merged. Note that the pairs of filensin/phakinin (A) and Fil(30–416)/phakinin (B) formed sinuous and mesh‐like filaments (yellow or yellowish green), while sometimes a few aggregates (yellow or yellowish green) were also observed in the same cell. In contrast, the Fil(30–369)/phakinin pair (C) gave aggregates (yellow or yellowish green). Single expression of phakinin in a cell gave aggregates (red). Scale bars: 20 μm.
**Fig. S2.** Expression of fluorescent protein‐tagged rat filensin, filensin fragments, and phakinin in MCF‐7 cells. Pairs of filensin/phakinin (A), Fil(30–416)/phakinin (B) and Fil(30–369)/phakinin (C) were transiently expressed in MCF‐7 cells. Fluorescence images of the filensin proteins tagged with AcGFP1 (green), phakinin tagged with mCherry (red), and nuclei stained with DAPI (blue) were merged. Note that the pairs of filensin/phakinin (A) and Fil(30–416)/phakinin (B) formed sinuous filaments (yellow or yellowish green). In contrast, the Fil(30–369)/phakinin pair (C) gave aggregates (yellow or yellowish green). Single expression of phakinin in a cell gave aggregates (red). Scale bars: 20 μm.

**Table S1.** Proportion (%) of SW‐13 cells containing filaments and/or aggregates.

## Data Availability

The data that support the findings of this study are contained within the article or [Link], [Link].
