# Supplementary material for: Effects of truncations in the N‐ and C‐terminal domains of filensin on filament formation with phakinin in cell‐free conditions and cultured cells
Source: FEBS Open Bio. 2023 Aug 30;13(11):1990–2004. doi: 10.1002/2211-5463.13700 (PMC10626283; doi:10.1002/2211-5463.13700)
Supplement: Supplementary file 1 — Fig. S1. Expression of fluorescent protein‐tagged rat filensin, filensin fragments, and phakinin in SW‐13 cells. Pairs of filensin/phakinin (A), Fil(30–416)/phakinin (B) and Fil(30–369)/phakinin (C) were transiently expressed in SW‐13 cells. Fluorescence images of the filensin proteins tagged with AcGFP1 (green), phakinin tagged with mCherry (red), and nuclei stained with DAPI (blue) were merged. Note that the pairs of filensin/phakinin (A) and Fil(30–416)/phakinin (B) formed sinuous and mesh‐like filaments (yellow or yellowish green), while sometimes a few aggregates (yellow or yellowish green) were also observed in the same cell. In contrast, the Fil(30–369)/phakinin pair (C) gave aggregates (yellow or yellowish green). Single expression of phakinin in a cell gave aggregates (red). Scale bars: 20 μm. Fig. S2. Expression of fluorescent protein‐tagged rat filensin, filensin fragments, and phakinin in MCF‐7 cells. Pairs of filensin/phakinin (A), Fil(30–416)/phakinin (B) and Fil(30–369)/phakinin (C) were transiently expressed in MCF‐7 cells. Fluorescence images of the filensin proteins tagged with AcGFP1 (green), phakinin tagged with mCherry (red), and nuclei stained with DAPI (blue) were merged. Note that the pairs of filensin/phakinin (A) and Fil(30–416)/phakinin (B) formed sinuous filaments (yellow or yellowish green). In contrast, the Fil(30–369)/phakinin pair (C) gave aggregates (yellow or yellowish green). Single expression of phakinin in a cell gave aggregates (red). Scale bars: 20 μm. [file FEB4-13-1990-s002.pdf]

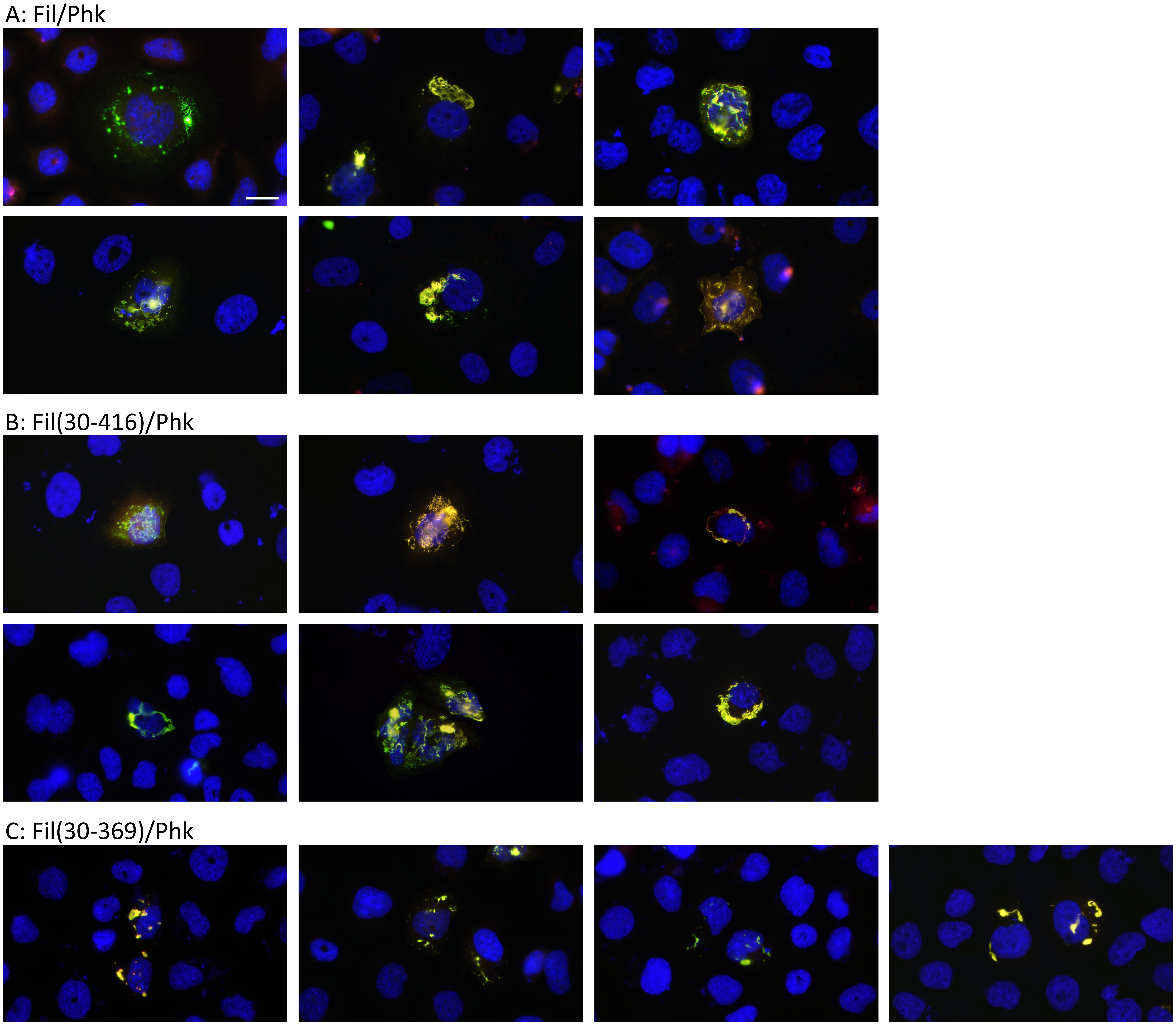

Supplementary Figure S1. Expression of fluorescent protein-tagged rat filensin, filensin fragments, and phakinin in SW-13 cells. Pairs of filensin/phakinin (A), Fil(30-416)/phakinin (B) and Fil(30-369)/phakinin (C) were transiently expressed in SW-13 cells. Fluorescence images of the filensin proteins tagged with AcGFP1 (green), phakinin tagged with mCherry (red) and nuclei stained with DAPI (blue) were merged. Note that the pairs of filensin/phakinin (A) and Fil(30-416)/phakinin (B) formed sinuous and mesh-like filaments (yellow or yellowish green), while sometimes a few aggregates (yellow or yellowish green) were also observed in the same cell. In contrast, the Fil(30-369)/phakinin pair (C) gave aggregates (yellow or yellowish green). Single expression of phakinin in a cell gave aggregates (red). Scale bars: 20 μm.

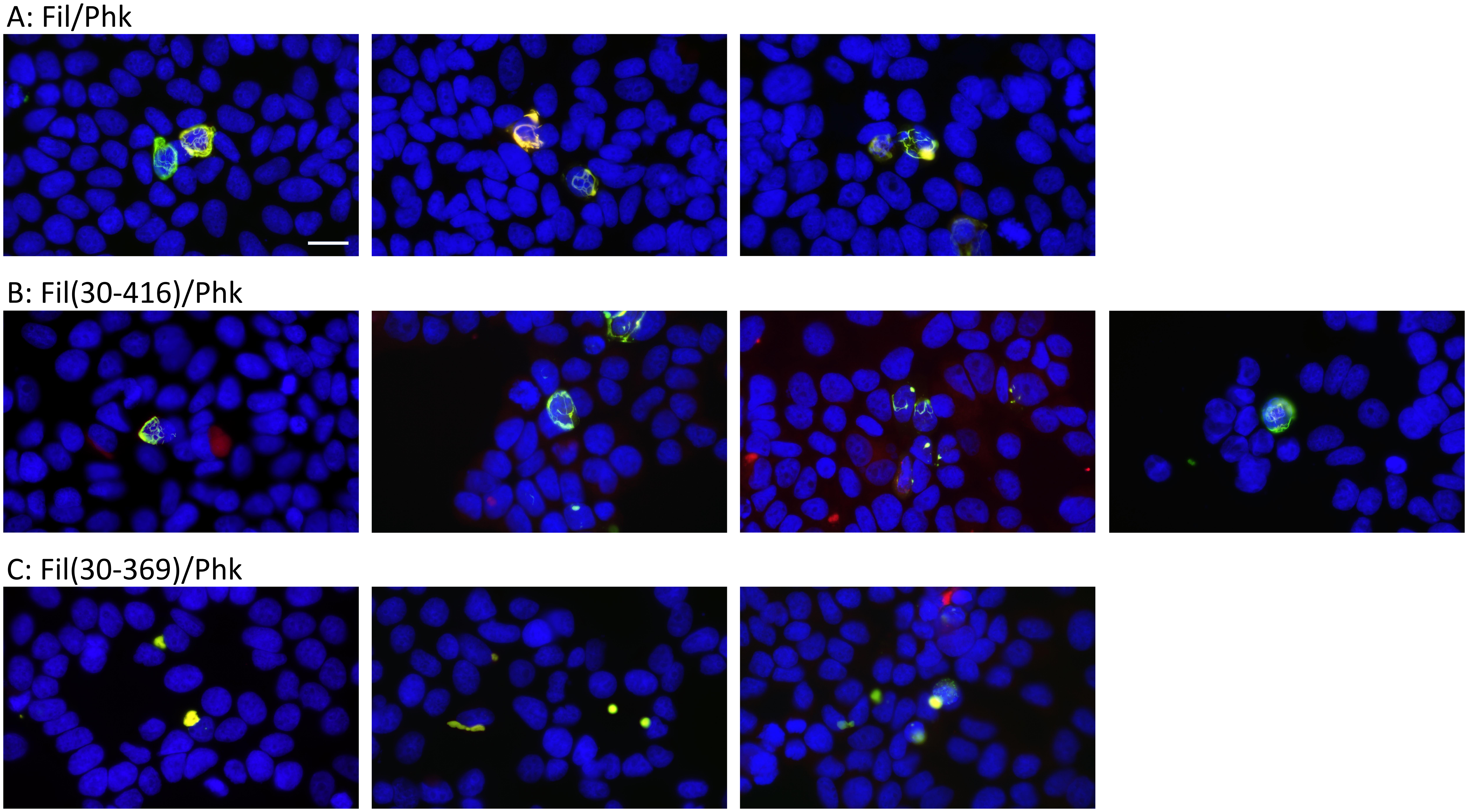

Supplementary Figure S2. Expression of fluorescent protein-tagged rat filensin, filensin fragments, and phakinin in MCF-7 cells. Pairs of filensin/phakinin (A), Fil(30-416)/phakinin (B) and Fil(30-369)/phakinin (C) were transiently expressed in MCF-7 cells. Fluorescence images of the filensin proteins tagged with AcGFP1 (green), phakinin tagged with mCherry (red) and nuclei stained with DAPI (blue) were merged. Note that the pairs of filensin/phakinin (A) and Fil(30-416)/phakinin (B) formed sinuous filaments (yellow or yellowish green). In contrast, the Fil(30-369)/phakinin pair (C) gave aggregates (yellow or yellowish green). Single expression of phakinin in a cell gave aggregates (red). Scale bars: 20 μm.
