# Supplementary material for: Effects of truncations in the N‐ and C‐terminal domains of filensin on filament formation with phakinin in cell‐free conditions and cultured cells
Source: FEBS Open Bio. 2023 Aug 30;13(11):1990–2004. doi: 10.1002/2211-5463.13700 (PMC10626283; doi:10.1002/2211-5463.13700)
Supplement: Supplementary file 2 — Table S1. Proportion (%) of SW‐13 cells containing filaments and/or aggregates. [file FEB4-13-1990-s001.pdf]

Supplementary Table S1. Proportion (%) of SW-13 cells containing filaments and/or aggregates.

Protein pair	Filaments	Filaments + Aggregates	Aggregates
Filensin/Phakinin (n = 58)	82	17	1
Fil(30-416)/Phakinin (n = 72)	82	15	3
Fil(30-369)/Phakinin (n = 141)	–	9	91

(n: counted cell number)
